# The genome sequence of the White-legged damselfly, *Platycnemis pennipes *(Pallas, 1771)

**DOI:** 10.12688/wellcomeopenres.19772.1

**Published:** 2023-07-25

**Authors:** Benjamin W. Price, E. Louise Allan, Natural History Museum Genome Acquisition Lab, Darwin Tree of Life Barcoding collective, Wellcome Sanger Institute Tree of Life programme, Wellcome Sanger Institute Scientific Operations: DNA Pipelines collective, Tree of Life Core Informatics collective, Darwin Tree of Life Consortium

**Affiliations:** 1 Natural History Museum, London, England, UK

**Keywords:** Platycnemis pennipes, White-legged damselfly, genome sequence, chromosomal, Odonata

## Abstract

We present a genome assembly from an individual male *Platycnemis pennipes* (the White-legged damselfly; Arthropoda; Insecta; Odonata; Platycnemididae). The genome sequence is 1793.3 megabases in span. Most of the assembly is scaffolded into 13 chromosomal pseudomolecules, including the X sex chromosome. The mitochondrial genome has also been assembled and is 15.42 kilobases in length.

## Species taxonomy

Eukaryota; Metazoa; Eumetazoa; Bilateria; Protostomia; Ecdysozoa; Panarthropoda; Arthropoda; Mandibulata; Pancrustacea; Hexapoda; Insecta; Dicondylia; Pterygota; Palaeoptera; Odonata; Zygoptera; Coenagrionoidea; Platycnemididae; Platycnemis (Pallas, 1771) (NCBI:txid126231).

## Background

The White-legged damselfly, *Platycnemis pennipes*, occurs throughout most of western and central Europe, with a southern distribution in England and Wales. It is the only species in the family Platycnemididae resident in the UK. It is found mainly along slow-flowing lowland streams and rivers, though it can sometimes be found on canals or ponds ( Brooks & Cham, 2014). The species is vulnerable to pollution, and especially vulnerable to physical disturbance of its bankside habitats ( Corbet & Brooks, 2008). Unusual among non-demoiselle damselflies, males of the white-legged damselfly use their white legs in courtship displays for females and threat displays to other males ( Brooks & Cham, 2014). Once mated, tandem pairs will often congregate for oviposition ( Martens, 1996). Adults are on the wing from late May to mid-August.

The genome of *P. pennipes* was sequenced as part of the Darwin Tree of Life Project, a collaborative effort to sequence all named eukaryotic species in the Atlantic Archipelago of Britain and Ireland. Here we present a chromosomally complete genome sequence for *Platycnemis pennipes*, based on one male specimen from Hever Castle, England.

## Genome sequence report

**Figure 1.**
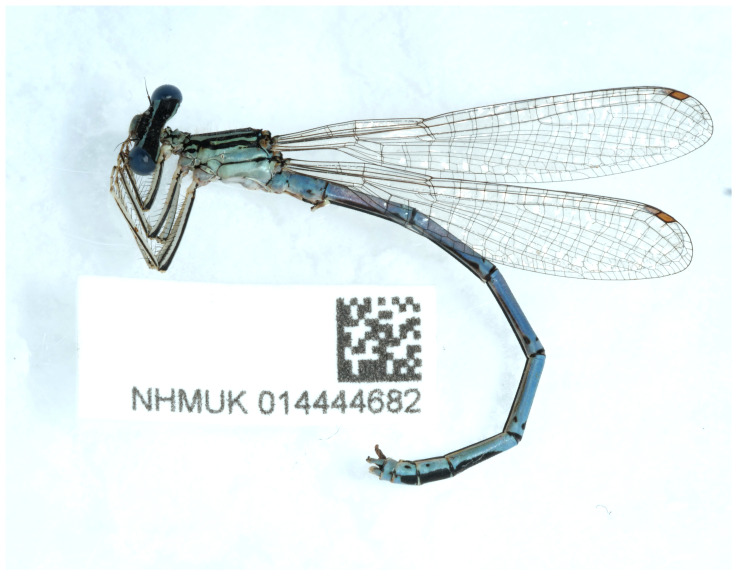
Photograph of the *Platycnemis pennipes* (ioPlaPenn1) specimen used for genome sequencing.

The genome was sequenced from one male *Platycnemis pennipes* ( Figure 1) collected from Hever Castle, England (51.19, 0.12). A total of 29-fold coverage in Pacific Biosciences single-molecule HiFi long was generated. Primary assembly contigs were scaffolded with chromosome conformation Hi-C data. Manual assembly curation corrected 37 missing joins or mis-joins and removed 12 haplotypic duplications, reducing the assembly length by 0.58% and the scaffold number by 5.43%.

**Figure 2.**
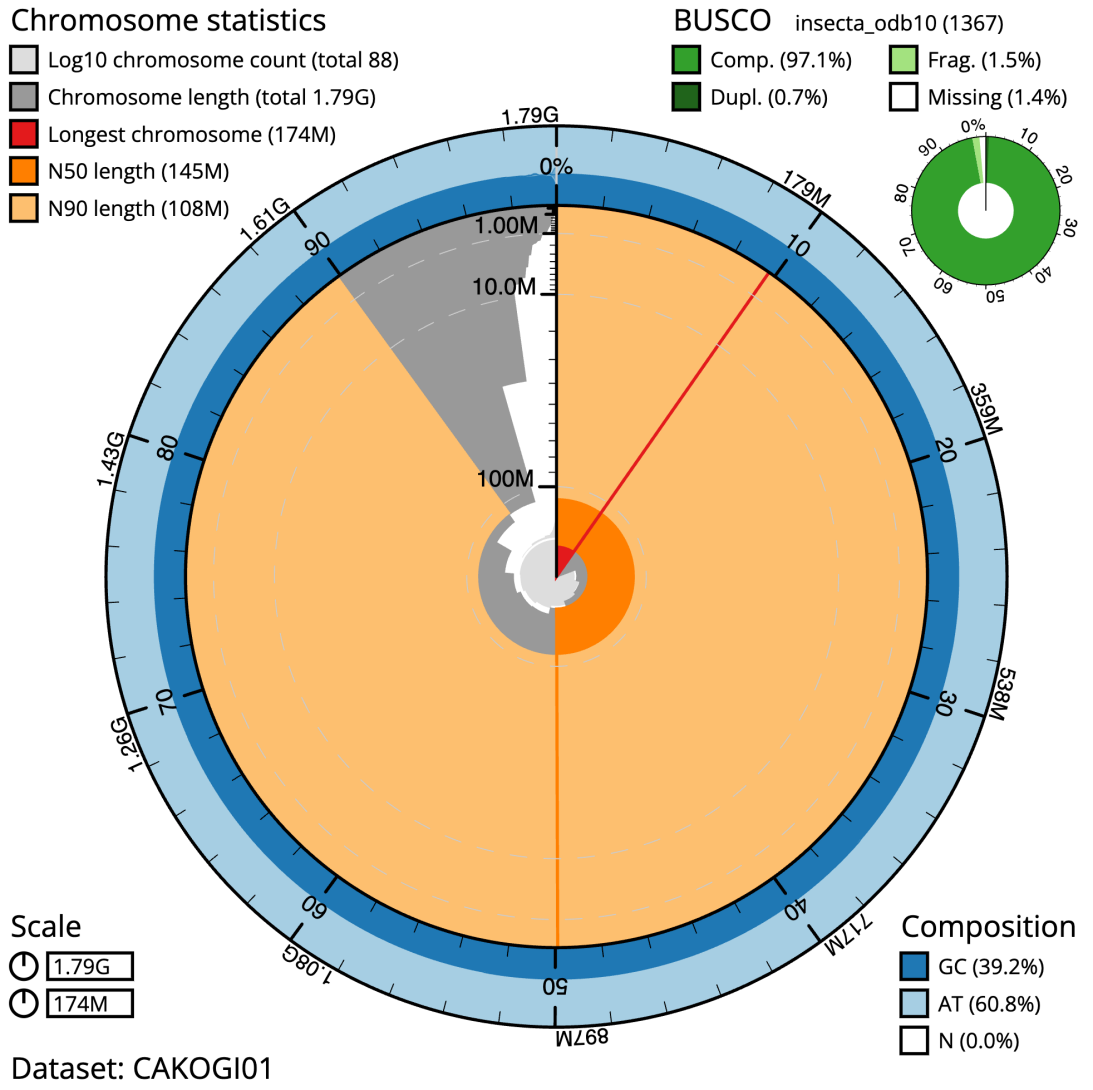
Genome assembly of *Platycnemis pennipes*, ioPlaPenn1.1: metrics. The BlobToolKit Snailplot shows N50 metrics and BUSCO gene completeness. The main plot is divided into 1,000 size-ordered bins around the circumference with each bin representing 0.1% of the 1,793,356,121 bp assembly. The distribution of scaffold lengths is shown in dark grey with the plot radius scaled to the longest scaffold present in the assembly (174,044,382 bp, shown in red). Orange and pale-orange arcs show the N50 and N90 scaffold lengths (144,837,943 and 108,313,761 bp), respectively. The pale grey spiral shows the cumulative scaffold count on a log scale with white scale lines showing successive orders of magnitude. The blue and pale-blue area around the outside of the plot shows the distribution of GC, AT and N percentages in the same bins as the inner plot. A summary of complete, fragmented, duplicated and missing BUSCO genes in the insecta_odb10 set is shown in the top right. An interactive version of this figure is available at https://blobtoolkit.genomehubs.org/view/ioPlaPenn1.1/dataset/CAKOGI01/snail.

The final assembly has a total length of 1793.3 Mb in 87 sequence scaffolds with a scaffold N50 of 144.8 Mb ( Table 1). Most (99.84%) of the assembly sequence was assigned to 13 chromosomal-level scaffolds, representing 12 autosomes and the X sex chromosome. Chromosome-scale scaffolds confirmed by the Hi-C data are named in order of size ( Figure 2– Figure 5; Table 2). The specimen was assigned as an XO male, since there was half-coverage of the X chromosome on the Hi-C map, with Hi-C data from a female specimen. While not fully phased, the assembly deposited is of one haplotype. Contigs corresponding to the second haplotype have also been deposited. The mitochondrial genome was also assembled and can be found as a contig within the multifasta file of the genome submission.

**Table 1.** Genome data for *Platycnemis pennipes*, ioPlaPenn1.1.

Project accession data		
Assembly identifier	ioPlaPenn1.1	
Species	*Platycnemis pennipes*	
Specimen	ioPlaPenn1	
NCBI taxonomy ID	126231	
BioProject	PRJEB50884	
BioSample ID	SAMEA9065986	
Isolate information	ioPlaPenn1, male: thorax (DNA sequencing) ioPlaPenn2, female: head (Hi-C scaffolding) ioPlaPenn3, male: thorax (RNA sequencing)	
Assembly metrics *		*Benchmark*
Consensus quality (QV)	66.4	*≥ 50*
*k*-mer completeness	100%	*≥ 95%*
BUSCO **	C:97.1%[S:96.4%,D:0.7%],F:1.5%,M:1.4%,n:1,367	*C ≥ 95%*
Percentage of assembly mapped to chromosomes	99.84%	*≥ 95%*
Sex chromosomes	X chromosome	*localised homologous pairs*
Organelles	Mitochondrial genome assembled	*complete single alleles*
Raw data accessions		
PacificBiosciences SEQUEL II	ERR8705869, ERR8705870, ERR8705871	
Hi-C Illumina	ERR8702792	
PolyA RNA-Seq Illumina	ERR10123681	
Genome assembly		
Assembly accession	GCA_933228895.1	
*Accession of alternate haplotype*	GCA_933228905.1	
Span (Mb)	1,793.3	
Number of contigs	252	
Contig N50 length (Mb)	18.2	
Number of scaffolds	87	
Scaffold N50 length (Mb)	144.8	
Longest scaffold (Mb)	174.0	

* Assembly metric benchmarks are adapted from column VGP-2020 of “Table 1: Proposed standards and metrics for defining genome assembly quality” from ( Rhie *et al.*, 2021). ** BUSCO scores based on the insecta_odb10 BUSCO set using v5.3.2. C = complete [S = single copy, D = duplicated], F = fragmented, M = missing, n = number of orthologues in comparison. A full set of BUSCO scores is available at https://blobtoolkit.genomehubs.org/view/ioPlaPenn1.1/dataset/CAKOGI01/busco.

**Figure 3.**
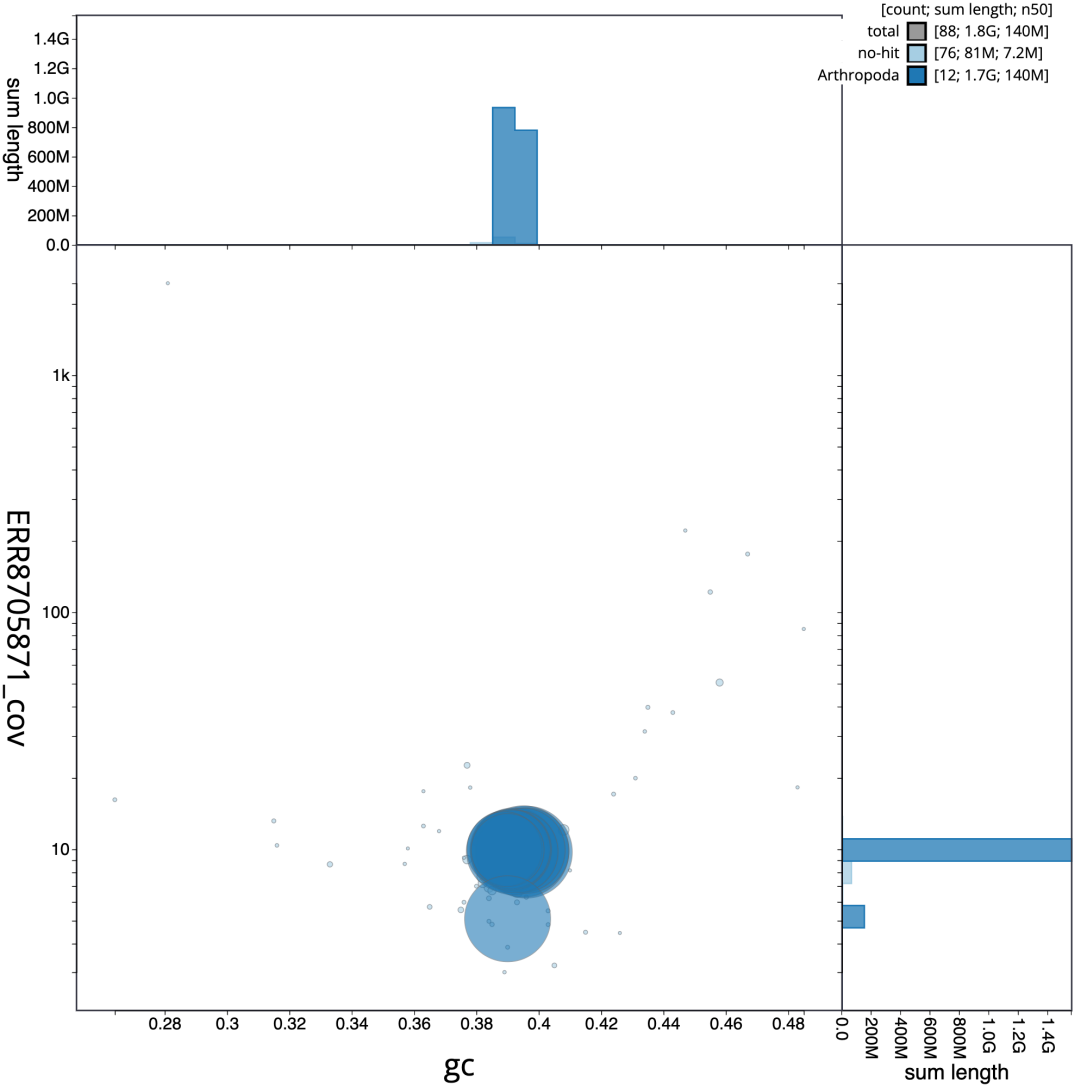
Genome assembly of *Platycnemis pennipes*, ioPlaPenn1.1: BlobToolKit GC-coverage plot. Scaffolds are coloured by phylum. Circles are sized in proportion to scaffold length. Histograms show the distribution of scaffold length sum along each axis. An interactive version of this figure is available at https://blobtoolkit.genomehubs.org/view/ioPlaPenn1.1/dataset/CAKOGI01/blob.

**Figure 4.**
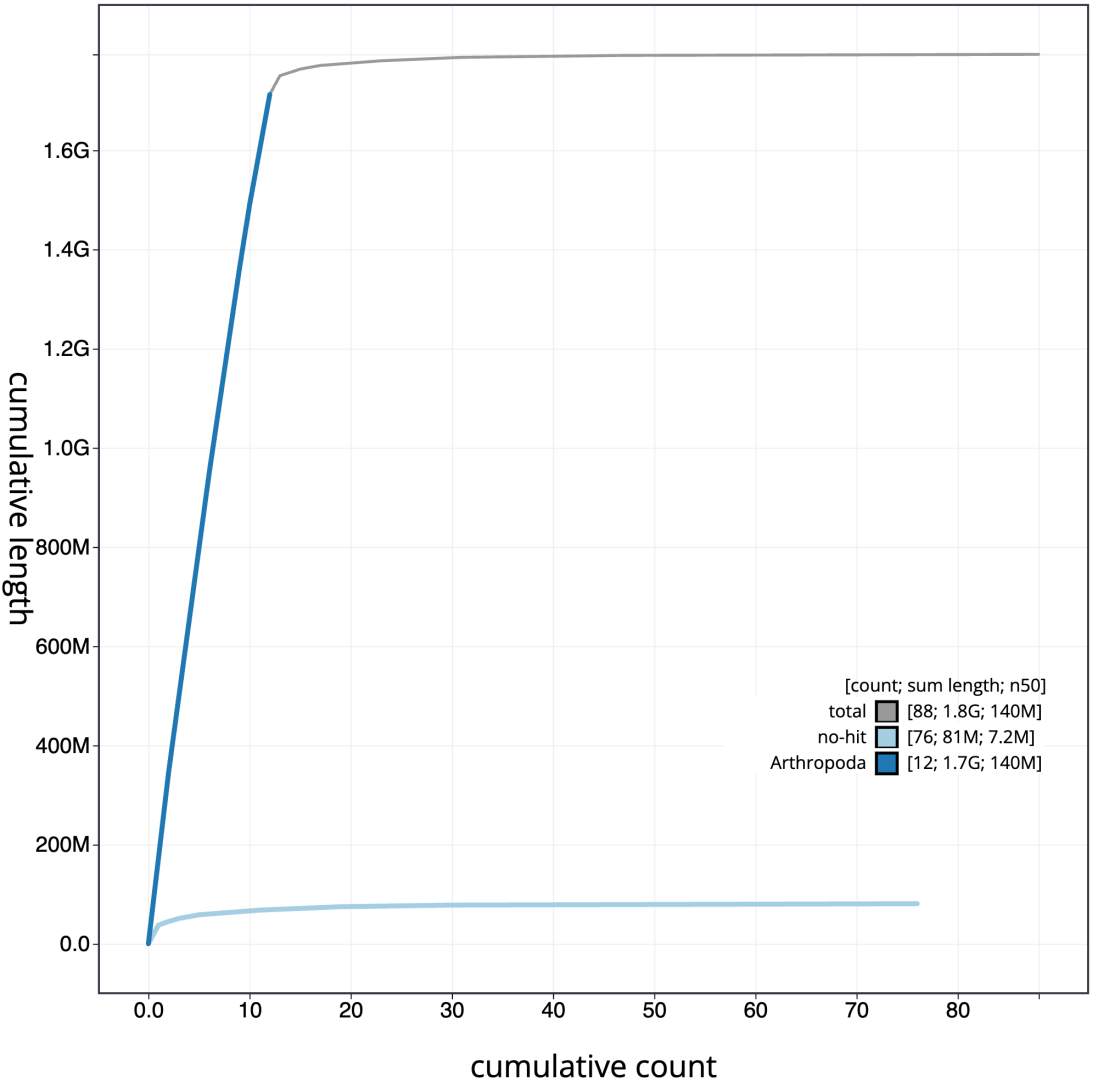
Genome assembly of *Platycnemis pennipes*, ioPlaPenn1.1: BlobToolKit cumulative sequence plot. The grey line shows cumulative length for all scaffolds. Coloured lines show cumulative lengths of scaffolds assigned to each phylum using the buscogenes taxrule. An interactive version of this figure is available at https://blobtoolkit.genomehubs.org/view/ioPlaPenn1.1/dataset/CAKOGI01/cumulative.

**Figure 5.**
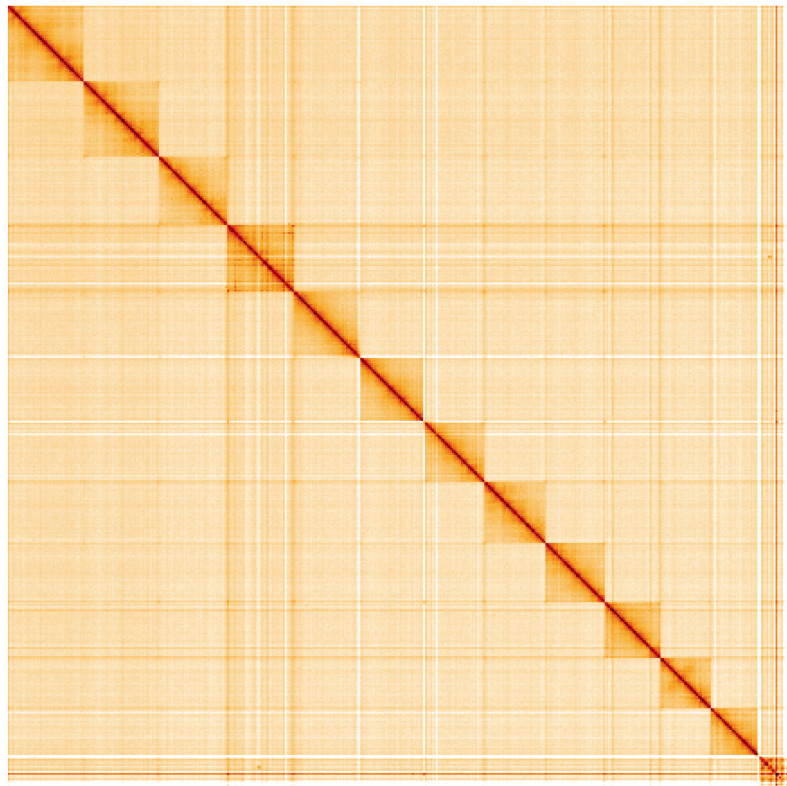
Genome assembly of *Platycnemis pennipes*, ioPlaPenn1.1: Hi-C contact map of the ioPlaPenn1.1 assembly, visualised using HiGlass. Chromosomes are shown in order of size from left to right and top to bottom. An interactive version of this figure may be viewed at https://genome-note-higlass.tol.sanger.ac.uk/l/?d=HvZd4tspTCKewtlkoJJ4dQ.

**Table 2.** Chromosomal pseudomolecules in the genome assembly of *Platycnemis pennipes*, ioPlaPenn1.

INSDC accession	Chromosome	Length (Mb)	GC%
OW121847.1	1	174.04	39.5
OW121848.1	2	171.77	39.5
OW121849.1	3	155.1	39.0
OW121850.1	4	150.02	39.5
OW121851.1	5	144.84	39.5
OW121852.1	6	139.34	39.0
OW121853.1	7	138.62	39.5
OW121854.1	8	135.49	39.0
OW121855.1	9	128.16	39.0
OW121856.1	10	114.91	39.0
OW121857.1	11	108.31	39.0
OW121858.1	12	37.71	39.0
OW121846.1	X	151.92	39.0
OW121859.1	MT	0.02	28.0

The estimated Quality Value (QV) of the final assembly is 66.4 with *k*-mer completeness of 100%, and the assembly has a BUSCO v5.3.2 completeness of 97.1% (single = 96.4%, duplicated = 0.7%), using the insecta_odb10 reference set ( *n* = 1,367).

Metadata for specimens, spectral estimates, sequencing runs, contaminants and pre-curation assembly statistics can be found at https://links.tol.sanger.ac.uk/species/126231.

## Methods

### Sample acquisition and nucleic acid extraction

The data presented here were derived from three *Platycnemis pennipes* specimens collected from the estate grounds at Hever Castle, England (latitude 51.19, longitude 0.12) on 2020-08-27 using an aerial net. The specimens were collected by Benjamin Price and Louise Allan (Natural History Museum) and identified by Benjamin Price. The specimens were preserved in liquid nitrogen. The specimen used for DNA sequencing was a male (specimen ID NHMUK014444682, ToLID ioPlaPenn1), a female specimen (specimen ID NHMUK014444686, ToLID ioPlaPenn2) was used for Hi-C scaffolding, and another male specimen (specimen ID NHMUK014444687, ToLID ioPlaPenn3) was used for RNA sequencing.

DNA was extracted at the Tree of Life laboratory, Wellcome Sanger Institute (WSI). The ioPlaPenn1 sample was weighed and dissected on dry ice with tissue set aside for Hi-C sequencing. Thorax tissue was disrupted using a Nippi Powermasher fitted with a BioMasher pestle. High molecular weight (HMW) DNA was extracted using the Qiagen MagAttract HMW DNA extraction kit. HMW DNA was sheared into an average fragment size of 12–20 kb in a Megaruptor 3 system with speed setting 30. Sheared DNA was purified by solid-phase reversible immobilisation using AMPure PB beads with a 1.8X ratio of beads to sample to remove the shorter fragments and concentrate the DNA sample. The concentration of the sheared and purified DNA was assessed using a Nanodrop spectrophotometer and Qubit Fluorometer and Qubit dsDNA High Sensitivity Assay kit. Fragment size distribution was evaluated by running the sample on the FemtoPulse system.

RNA was extracted from thorax tissue of ioPlaPenn3 in the Tree of Life Laboratory at the WSI using TRIzol, according to the manufacturer’s instructions. RNA was then eluted in 50 μl RNAse-free water and its concentration assessed using a Nanodrop spectrophotometer and Qubit Fluorometer using the Qubit RNA Broad-Range (BR) Assay kit. Analysis of the integrity of the RNA was done using Agilent RNA 6000 Pico Kit and Eukaryotic Total RNA assay.

### Sequencing

Pacific Biosciences HiFi circular consensus DNA sequencing libraries were constructed according to the manufacturers’ instructions. Poly(A) RNA-Seq libraries were constructed using the NEB Ultra II RNA Library Prep kit. DNA and RNA sequencing was performed by the Scientific Operations core at the WSI on Pacific Biosciences SEQUEL II (HiFi) and Illumina NovaSeq 6000 (RNA-Seq) instruments. Hi-C data were also generated from head tissue of ioPlaPenn2 using the Arima2 kit and sequenced on the Illumina NovaSeq 6000 instrument.

### Genome assembly, curation and evaluation

Assembly was carried out with Hifiasm ( Cheng *et al.*, 2021) and haplotypic duplication was identified and removed with purge_dups ( Guan *et al.*, 2020). The assembly was then scaffolded with Hi-C data ( Rao *et al.*, 2014) using YaHS ( Zhou *et al.*, 2023). The assembly was checked for contamination and corrected using the gEVAL system ( Chow *et al.*, 2016) as described previously ( Howe *et al.*, 2021). Manual curation was performed using gEVAL, HiGlass ( Kerpedjiev *et al.*, 2018) and Pretext ( Harry, 2022). The mitochondrial genome was assembled using MitoHiFi ( Uliano-Silva *et al.*, 2022), which runs MitoFinder ( Allio *et al.*, 2020) or MITOS ( Bernt *et al.*, 2013) and uses these annotations to select the final mitochondrial contig and to ensure the general quality of the sequence.

A Hi-C map for the final assembly was produced using bwa-mem2 ( Vasimuddin *et al.*, 2019) in the Cooler file format ( Abdennur & Mirny, 2020). To assess the assembly metrics, the *k*-mer completeness and QV consensus quality values were calculated in Merqury ( Rhie *et al.*, 2020). This work was done using Nextflow ( Di Tommaso *et al.*, 2017) DSL2 pipelines “sanger-tol/readmapping” ( Surana *et al.*, 2023a) and “sanger-tol/genomenote” ( Surana *et al.*, 2023b). The genome was analysed within the BlobToolKit environment ( Challis *et al.*, 2020) and BUSCO scores ( Manni *et al.*, 2021; Simão *et al.*, 2015) were calculated.

Table 3 contains a list of relevant software tool versions and sources.

**Table 3.** Software tools: versions and sources.

Software tool	Version	Source
BlobToolKit	3.5.3	https://github.com/blobtoolkit/blobtoolkit
BUSCO	5.3.2	https://gitlab.com/ezlab/busco
gEVAL	N/A	https://geval.org.uk/
Hifiasm	0.16.1-r375	https://github.com/chhylp123/hifiasm
HiGlass	1.11.6	https://github.com/higlass/higlass
Merqury	MerquryFK	https://github.com/thegenemyers/MERQURY.FK
MitoHiFi	2	https://github.com/marcelauliano/MitoHiFi
PretextView	0.2	https://github.com/wtsi-hpag/PretextView
purge_dups	1.2.3	https://github.com/dfguan/purge_dups
sanger-tol/genomenote	v1.0	https://github.com/sanger-tol/genomenote
sanger-tol/readmapping	1.1.0	https://github.com/sanger-tol/readmapping/tree/1.1.0
YaHS	yahs-1.1.91eebc2	https://github.com/c-zhou/yahs

### Wellcome Sanger Institute – Legal and Governance

The materials that have contributed to this genome note have been supplied by a Darwin Tree of Life Partner. The submission of materials by a Darwin Tree of Life Partner is subject to the **‘Darwin Tree of Life Project Sampling Code of Practice’**, which can be found in full on the Darwin Tree of Life website here. By agreeing with and signing up to the Sampling Code of Practice, the Darwin Tree of Life Partner agrees they will meet the legal and ethical requirements and standards set out within this document in respect of all samples acquired for, and supplied to, the Darwin Tree of Life Project. 

Further, the Wellcome Sanger Institute employs a process whereby due diligence is carried out proportionate to the nature of the materials themselves, and the circumstances under which they have been/are to be collected and provided for use. The purpose of this is to address and mitigate any potential legal and/or ethical implications of receipt and use of the materials as part of the research project, and to ensure that in doing so we align with best practice wherever possible. The overarching areas of consideration are: 

Ethical review of provenance and sourcing of the material

Legality of collection, transfer and use (national and international)

Each transfer of samples is further undertaken according to a Research Collaboration Agreement or Material Transfer Agreement entered into by the Darwin Tree of Life Partner, Genome Research Limited (operating as the Wellcome Sanger Institute), and in some circumstances other Darwin Tree of Life collaborators.

## Data Availability

European Nucleotide Archive: *Platycnemis pennipes*. Accession number PRJEB50884; https://identifiers.org/ena.embl/PRJEB50884. ( Wellcome Sanger Institute, 2022) The genome sequence is released openly for reuse. The *Platycnemis pennipes* genome sequencing initiative is part of the Darwin Tree of Life (DToL) project. All raw sequence data and the assembly have been deposited in INSDC databases. The genome will be annotated using available RNA-Seq data and presented through the Ensembl pipeline at the European Bioinformatics Institute. Raw data and assembly accession identifiers are reported in Table 1.

## Author information

Members of the Natural History Museum Genome Acquisition Lab are listed here: https://doi.org/10.5281/zenodo.4790042.

Members of the Darwin Tree of Life Barcoding collective are listed here: https://doi.org/10.5281/zenodo.4893703.

Members of the Wellcome Sanger Institute Tree of Life programme are listed here: https://doi.org/10.5281/zenodo.4783585.

Members of Wellcome Sanger Institute Scientific Operations: DNA Pipelines collective are listed here: https://doi.org/10.5281/zenodo.4790455.

Members of the Tree of Life Core Informatics collective are listed here: https://doi.org/10.5281/zenodo.5013541.

Members of the Darwin Tree of Life Consortium are listed here: https://doi.org/10.5281/zenodo.4783558.

## References

[ref-1] Abdennur N Mirny LA : Cooler: Scalable storage for Hi-C data and other genomically labeled arrays. *Bioinformatics.* 2020;36(1):311–316. 10.1093/bioinformatics/btz540 31290943 PMC8205516

[ref-2] Allio R Schomaker‐Bastos A Romiguier J : MitoFinder: Efficient automated large-scale extraction of mitogenomic data in target enrichment phylogenomics. *Mol Ecol Resour.* 2020;20(4):892–905. 10.1111/1755-0998.13160 32243090 PMC7497042

[ref-3] Bernt M Donath A Jühling F : MITOS: Improved *de novo* metazoan mitochondrial genome annotation. *Mol Phylogenet Evol.* 2013;69(2):313–319. 10.1016/j.ympev.2012.08.023 22982435

[ref-4] Brooks S Cham S : Field Guide to the Dragonflies & Damselflies of Great Britain and Ireland. Oxford: British Wildlife Publishing Ltd,2014.

[ref-5] Challis R Richards E Rajan J : BlobToolKit - interactive quality assessment of genome assemblies. *G3 (Bethesda).* 2020;10(4):1361–1374. 10.1534/g3.119.400908 32071071 PMC7144090

[ref-6] Cheng H Concepcion GT Feng X : Haplotype-resolved *de novo* assembly using phased assembly graphs with hifiasm. *Nat Methods.* 2021;18(2):170–175. 10.1038/s41592-020-01056-5 33526886 PMC7961889

[ref-7] Chow W Brugger K Caccamo M : gEVAL — a web-based browser for evaluating genome assemblies. *Bioinformatics.* 2016;32(16):2508–2510. 10.1093/bioinformatics/btw159 27153597 PMC4978925

[ref-8] Corbet PS Brooks SJ : Dragonflies. London: CollinsNew Naturalist Library,2008.

[ref-9] Di Tommaso P Chatzou M Floden EW : Nextflow enables reproducible computational workflows. *Nat Biotechnol.* 2017;35(4):316–319. 10.1038/nbt.3820 28398311

[ref-10] Guan D McCarthy SA Wood J : Identifying and removing haplotypic duplication in primary genome assemblies. *Bioinformatics.* 2020;36(9):2896–2898. 10.1093/bioinformatics/btaa025 31971576 PMC7203741

[ref-11] Harry E : PretextView (Paired REad TEXTure Viewer): A desktop application for viewing pretext contact maps.2022; [Accessed 19 October 2022]. Reference Source

[ref-12] Howe K Chow W Collins J : Significantly improving the quality of genome assemblies through curation. *GigaScience.* Oxford University Press,2021;10(1): giaa153. 10.1093/gigascience/giaa153 33420778 PMC7794651

[ref-13] Kerpedjiev P Abdennur N Lekschas F : HiGlass: web-based visual exploration and analysis of genome interaction maps. *Genome Biol.* 2018;19(1): 125. 10.1186/s13059-018-1486-1 30143029 PMC6109259

[ref-14] Manni M Berkeley MR Seppey M : BUSCO update: Novel and streamlined workflows along with broader and deeper phylogenetic coverage for scoring of eukaryotic, prokaryotic, and viral genomes. *Mol Biol Evol.* 2021;38(10):4647–4654. 10.1093/molbev/msab199 34320186 PMC8476166

[ref-15] Martens A : Die Federlibellen Europas.Platycnemididae. Heidelberg: Westarp Wissenschaften, Magdeburg & Spektrum Akademischer Verlag,1996.

[ref-16] Rao SSP Huntley MH Durand NC : A 3D map of the human genome at kilobase resolution reveals principles of chromatin looping. *Cell.* 2014;159(7):1665–1680. 10.1016/j.cell.2014.11.021 25497547 PMC5635824

[ref-17] Rhie A McCarthy SA Fedrigo O : Towards complete and error-free genome assemblies of all vertebrate species. *Nature.* 2021;592(7856):737–746. 10.1038/s41586-021-03451-0 33911273 PMC8081667

[ref-18] Rhie A Walenz BP Koren S : Merqury: Reference-free quality, completeness, and phasing assessment for genome assemblies. *Genome Biol.* 2020;21(1)245. 10.1186/s13059-020-02134-9 32928274 PMC7488777

[ref-19] Simão FA Waterhouse RM Ioannidis P : BUSCO: assessing genome assembly and annotation completeness with single-copy orthologs. *Bioinformatics.* 2015;31(19):3210–3212. 10.1093/bioinformatics/btv351 26059717

[ref-20] Surana P Muffato M Qi G : sanger-tol/readmapping: sanger-tol/readmapping v1.1.0 - Hebridean Black (1.1.0). *Zenodo* .2023a. 10.5281/zenodo.7755665

[ref-21] Surana P Muffato M Sadasivan Baby C : sanger-tol/genomenote (v1.0.dev). *Zenodo* .2023b. 10.5281/zenodo.6785935

[ref-22] Uliano-Silva M Ferreira JGRN Krasheninnikova K : MitoHiFi: a python pipeline for mitochondrial genome assembly from PacBio High Fidelity reads. *bioRxiv.* 2022. 10.1101/2022.12.23.521667 PMC10354987 37464285

[ref-23] Vasimuddin M Misra S Li H : Efficient Architecture-Aware Acceleration of BWA-MEM for Multicore Systems.In: *2019 IEEE Int Parallel Distrib Process Symp (IPDPS).* IEEE,2019;314–324. 10.1109/IPDPS.2019.00041

[ref-25] Wellcome Sanger Institute: The genome sequence of the White-legged damselfly, *Platycnemis pennipes* (Pallas, 1771). European Nucleotide Archive.[dataset], accession number PRJEB50884,2022.

[ref-24] Zhou C McCarthy SA Durbin R : YaHS: yet another Hi-C scaffolding tool. *Bioinformatics.* 2023;39(1): btac808. 10.1093/bioinformatics/btac808 36525368 PMC9848053

