# Supplementary material for: The genome sequence of the White-legged damselfly, Platycnemis pennipes (Pallas, 1771)
Source: Wellcome Open Res. 2023 Jul 25;8:320. [Version 1] doi: 10.12688/wellcomeopenres.19772.1 (PMC11099507; doi:10.12688/wellcomeopenres.19772.1)
Supplement: Peer review history

# Reviewer report: Reviewer response for version 1

**DOI:** 10.21956/wellcomeopenres.21899.r75671

**Published:** 2024-05-16

**Authors:** Rhys Parry

**Affiliations:** 1 The University of Queensland, Saint Lucia, Queensland, Australia

Price and Allan present the genome sequence of the White-legged damselfly, Platycnemis pennipes, assembled with two samples (one male, one female) and one male specimen for RNA sequencing.

 I have slight reservations about positive identification of the samples (male and female) and confidence of identification either through morphology or independent assembly of the mtDNA from both samples and then comparisons but it is probably unnecessary.

 The assembly appears to be of high quality with good BUSCO scores, the data is available, and the pipeline for assembly is sound.

Are sufficient details of methods and materials provided to allow replication by others?

Yes

Is the rationale for creating the dataset(s) clearly described?

Yes

Are the datasets clearly presented in a useable and accessible format?

Yes

Are the protocols appropriate and is the work technically sound?

Yes

Reviewer Expertise:

Metagenomics and genome assemblies of bacteria/viruses from environmental sources.

I confirm that I have read this submission and believe that I have an appropriate level of expertise to confirm that it is of an acceptable scientific standard.

# Reviewer report: Reviewer response for version 1

**DOI:** 10.21956/wellcomeopenres.21899.r75670

**Published:** 2024-04-05

**Authors:** Jeffrey Marcus

**Affiliations:** 1 University of Manitoba, Winnipeg, Manitoba, Canada

In this manuscript, the authors describe the sequencing and assembly of the *Platycnemis pennipes *genome using nucleic acids from 3 specimens collected in the UK.  The primary genome sequence assembly is from a single male specimen, from which proposed chromosomal pseudomolecule sequences were proposed for 12 autosomes, a sex chromosome, and a complete mitochondrial genome. A female specimen had DNA sequenced to assist in identifying the sex chromosomes in the assembly and to infer the sex determining mechanism in this species. Males appear to be XO and females XX in *P. pennipes.  *An additional male specimen was used for RNA sequencing, presumably to assist in identifying coding sequences in the assembled genome. Overall, this is a solid contribution to the scientific literature.

 Some suggestions to the authors:

1. Background:  “The White-legged damselfly, *Platycnemis pennipes*, occurs throughout most of western and central Europe, with a southern distribution in England and Wales.”  It is not clear from the text whether the authors are suggesting that England and Wales are the southern parts of the European distribution of this species or instead that they are suggesting that this species can be found in the southern parts of England and Wales in addition to portions of continental Europe.
2. Genome sequence report:  “The genome was sequenced from one male *Platycnemis pennipes…”*. The ID and ToLID numbers for the relevant specimen should be included at first mention.
3. Genome sequence report:  “The specimen was assigned as an XO male, since there was half-coverage of the X chromosome on the Hi-C map with Hi-C data from a female specimen.”  This is the first mention of the existence of a female specimen, let alone the sequencing of that specimen, in the document.  Consequently, this statement is confusing and is not clarified until 3 paragraphs later when this female specimen is described in additional detail in the Methods.
4. Figure 5. Numbering the chromosomes in this figure would be helpful to those trying to decipher it, either along the axes, or adjacent to the dark squares for each chromosome rendered by HiGlass.
5. I agree with Xiaohan Shu that additional description of the annotation process for the nuclear and mitochondrial genomes would be helpful to readers.  As it currently stands, the methods for annotation are clear, but not the results.

Are sufficient details of methods and materials provided to allow replication by others?

Yes

Is the rationale for creating the dataset(s) clearly described?

Yes

Are the datasets clearly presented in a useable and accessible format?

Partly

Are the protocols appropriate and is the work technically sound?

Yes

Reviewer Expertise:

Evolutionary biology of insects, phylogenomics

I confirm that I have read this submission and believe that I have an appropriate level of expertise to confirm that it is of an acceptable scientific standard, however I have significant reservations, as outlined above.

# Reviewer report: Reviewer response for version 1

**DOI:** 10.21956/wellcomeopenres.21899.r74488

**Published:** 2024-02-26

**Authors:** Xiaohan Shu

**Affiliations:** 1 Zhejiang University, Hangzhou, Zhejiang, China

This manuscript reports the chromosomal-level genome of the White-legged damselfly, *Platycnemis pennipes*, providing a new genomic resource for damselfly research. The authors also report the mitochondrial genome of *P. pennipes*. It is a good work. 

 Below are suggestions for modifications:

 1. The authors could provide annotation information for the assembled genome and the mitochondrial genome.

 2. In Figure 5, it would be beneficial to label each chromosome with its number. Additionally, placing the X chromosome at the end may be more logical.

Are sufficient details of methods and materials provided to allow replication by others?

Yes

Is the rationale for creating the dataset(s) clearly described?

Yes

Are the datasets clearly presented in a useable and accessible format?

Yes

Are the protocols appropriate and is the work technically sound?

Yes

Reviewer Expertise:

Insect genomics, Ecology & Evolution

I confirm that I have read this submission and believe that I have an appropriate level of expertise to confirm that it is of an acceptable scientific standard.
